# Febrile temperature activates the innate immune response by promoting aberrant influenza A virus RNA synthesis

**DOI:** 10.1126/sciadv.aeb2700

**Published:** 2026-01-02

**Authors:** Karishma Bisht, Daniel R. Weilandt, Caitlin H. Lamb, Elizaveta Elshina, Cameron Myhrvold, Aartjan J.W. te Velthuis

**Affiliations:** ^1^ Lewis Thomas Laboratory, Department of Molecular Biology, Princeton University, Princeton, NJ 08544, USA.; ^2^ Department of Chemistry, Princeton University, Princeton, NJ 08544, USA.; ^3^ Lewis-Sigler Institute of Integrative Genomics, Princeton University, Princeton, NJ 08544, USA.; ^4^ Ludwig Institute for Cancer Research, Princeton University, Princeton, NJ 08544, USA.; ^5^ Department of Chemical and Biological Engineering, Princeton University, Princeton, NJ 08544, USA.; ^6^ Omenn-Darling Bioengineering Institute, Princeton University, Princeton, NJ 08544, USA.

## Abstract

Fever during influenza A virus (IAV) infection is triggered by the innate immune response. Various factors contribute to this response, including IAV mini viral RNAs (mvRNA), which trigger RIG-I signaling when their replication and transcription are dysregulated by template loops (t-loops). It is presently not well understood whether the fever response to IAV infection affects subsequent viral replication and innate immune activation. Here, we show that IAV infection at temperatures that simulate fever leads to increased antiviral signaling in H1N1 and H3N2 infections. Mathematical modeling and experimental analyses reveal that differential IAV nucleoprotein and RNA polymerase production increase mvRNA and interferon production. Moreover, at the higher infection temperature, mvRNAs with dysregulating t-loops contribute most to the innate immune activation. We propose that fever during IAV infection can establish a positive feedback loop in which elevated aberrant RNA synthesis and innate immune activation can contribute to the dysregulation of cytokine production.

## Teaser

Fever promotes aberrant influenza A virus RNA synthesis and innate immune activation.

## INTRODUCTION

Influenza A viruses (IAVs) cause moderate to severe respiratory disease in seasonal epidemics and occasional pandemics (*1*). The level of activation and dysregulation of the innate immune response contributes to the outcome of IAV infection, particularly in infections with highly pathogenic avian or pandemic IAV strains (*2*, *3*). However, the processes involved are complex and still incompletely understood. Activation of the innate immune response is generally accepted to involve the binding of IAV RNA to host pathogen receptor retinoic acid–inducible gene I (RIG-I) (*4*–*6*). Once activated, RIG-I initiates the oligomerization of the mitochondrial antiviral signaling (MAVS) protein, triggering the phosphorylation of nuclear factor κB (NF-κB), interferon regulatory factor–3 (IRF-3), or IRF-7 and the subsequent expression of interferons (IFNs) type I and III and proinflammatory genes, including tumor necrosis factor (TNF) and interleukin-6 (IL-6) (*7*, *8*). In turn, IFNs drive signal transducers and activators of transcription 1 (STAT1) and STAT2 phosphorylation and the expression of IFN-stimulated genes (ISGs). Proinflammatory genes and ISGs have many roles in IAV infection, including recruitment of immune cells, activation of negative feedback loops, and triggering the febrile response that elevates the host temperature from ~37° to 38.5° to 41.0°C (*9*, *10*). While fever has been known to be a common and early IAV infection symptom for decades, its impact on viral replication and gene expression is understudied.

**Fig. 1.**
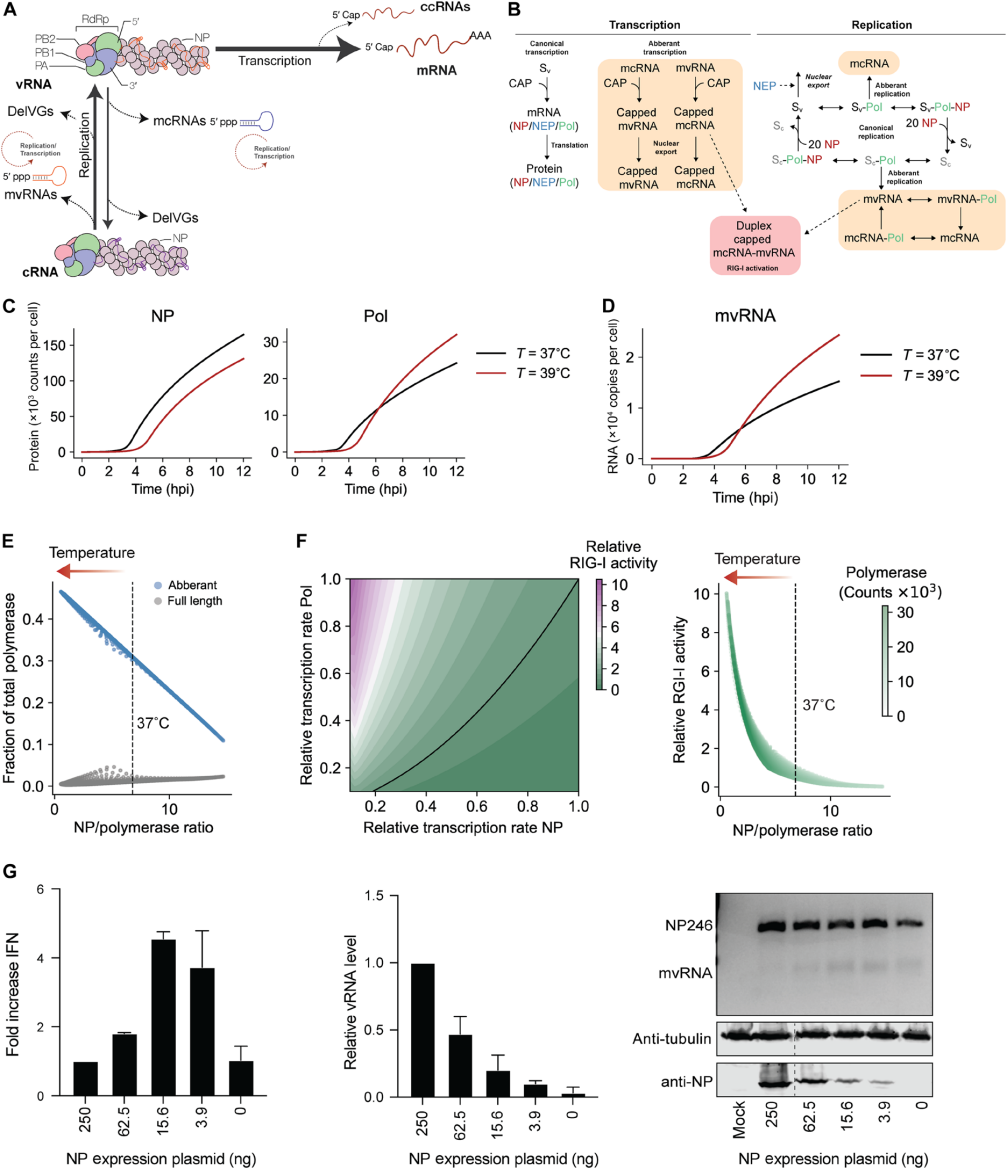
The single infection cycle model of IAV reveals impact of NP dynamics on innate immune activation. (**A**) Schematic of IAV replication and transcription during IAV infection. The vRNP consists of a copy of the vRNA-dependent RNA polymerase (RdRp), vRNA (orange), and NP (purple). The RdRp consists of three subunits: PB1 (blue), PA (green), and PB2 (pink). Canonical transcription of vRNA molecules produces mRNAs, while replication of vRNA produces cRNAs (purple). cRNA molecules are encapsidated into cRNPs. Noncanonical transcription of vRNAs can produce ccRNAs, while noncanonical replication of vRNA molecules can produce mcRNAs and DelVGs. Also, cRNA molecules can be replicated into mvRNAs and DelVGs. The mcRNAs and mvRNAs can be transcribed and replicated (dotted circles). (**B**) Schematic illustrating the processes and variables (see table S2) incorporated into the model. (**C**) Simulation of IAV infection cycle and prediction of NP and polymerase protein levels per cell. (**D**) Simulation of IAV infection cycle and prediction of mvRNA levels per cell. (**E**) Replication rate of aberrant and full-length products driven by the NP/polymerase ratio. (**F**) Heatmap on left shows RIG-I activity relative to 37°C, as function of NP and polymerase gene transcription. Plot on right shows RIG-I activity as function of the ratio between the NP and polymerase proteins that follows from the simulations in left plot. Arrow indicates shift in RIG-I activation after fever. (**G**) Analysis of *IFNB* promoter activity and vRNA levels using primer extension during the replication of a segment 5–based 246-nt RNA template by the WSN polymerases. NP mvRNA levels were analyzed by reverse transcription polymerase chain reaction (RT-PCR). NP expression was assessed by Western blot (bottom gel). *n* = 3 biologically independent experiments.

The IAV genome consists of eight segments of single-stranded, negative sense viral RNA (vRNA) that exist in the context of viral ribonucleoproteins (vRNPs) and are bound by at least 20 nucleoprotein (NP) molecules (*11*, *12*). The replication and transcription of the viral genome are complex processes that involve both viral and host factors (Fig. 1A). The IAV RNA polymerase first produces capped and polyadenylated mRNA molecules during IAV transcription. Next, the IAV RNA polymerase produces a complementary RNA (cRNA) that is subsequently copied into a new vRNA molecule as part of viral replication. In addition, the IAV RNA polymerase produces various aberrant or noncanonical RNA molecules, including deletion-containing viral genomes (DelVGs), mini vRNAs (mvRNAs), and capped cRNAs (ccRNAs) (Fig. 1A) (*13*–*17*). DelVGs and mvRNAs are diverse in sequence and lack internal gene sequences but retain the conserved 5′ and 3′ termini of full-length vRNA segments that act as viral promoter. DelVGs and mvRNAs can therefore be replicated and transcribed. The difference between the two molecules is that mvRNAs are sufficiently short (<125 nt) to be replicated and transcribed in the absence of viral NP and that a reduction in NP levels stimulates mvRNA formation. In contrast to mvRNAs and DelVGs, ccRNAs are IAV transcripts that contain a cap but lack a polyadenylate [poly(A)] tail (Fig. 1A) (*15*, *16*). Presently, the role of mvRNAs and ccRNAs is not fully understood. One role of mvRNAs and ccRNAs in infection is activation of RIG-I signaling. mvRNAs that contain a so-called template loop (t-loop) contribute most to RIG-I activation because the t-loop causes ccRNA formation during mvRNA transcription and RNA polymerase stalling during mvRNA replication, which together lead to the release of mvRNAs and the putative formation of mvRNA-ccRNA duplexes (*15*). While IAV replication and transcription have been captured with computational models previously, they have not been combined into a single model that also captures IAV aberrant RNA synthesis and innate immune signaling.

IAVs can infect cells of the upper and lower respiratory tract, and the viral replication machinery may thus experience different temperature environments during infection (*18*–*20*). In addition, the IAV replication machinery may experience a change in temperature when innate immune signaling activates the febrile response. Previous in vitro studies have shown that IAV RNA synthesis is altered when the incubation temperature is increased (*21*), but the impact of the temperature on IAV infection in cells is still poorly understood. A potential confounding factor in such a study is that simulation of the febrile response through heat shock will affect host cell gene expression, host posttranslational modifications, and viral enzymatic activity. These combinatorial effects make it difficult to measure how an increase in temperature affects the efficiency of IAV replication and transcription, and how putative aberrant RNAs produced under this condition affect the innate immune system. In vivo, the impact of temperature on IAV infection in small animal models is strongly dependent on the animal used. For instance, mice enter hypothermia or show no fever response following IAV infection (*22*), whereas ferrets do show a febrile response upon infection (*23*). Systematic insight into the dependency of host and viral factors on temperature and their modulation by temperature would help us better understand the role of the febrile response in IAV infection.

To explore the impact of temperature on the multiple viral processes and study the interactions among IAV replication, transcription, aberrant RNA synthesis, and innate immune activation, we used mathematical modeling to simulate a single infection cycle. Our modeling predicted that a change in infection temperature from 37° to 39°C would lead to dysregulated viral NP and polymerase acidic (PA) subunit levels and an increase in mvRNA production and innate immune signaling. This change in temperature simulates a mild fever response similar to the flu symptoms observed during an infection with seasonal and pandemic IAV strains. We subsequently designed experiments to verify our model and measure the effect of temperature on IAV RNA synthesis. Specifically, we acclimated cells to 37° or 39°C before infection or transfection to minimize the impact of heat shock and avoid differential gene expression between cells growing at either temperature. Overall, we find that our experimental results align with our model and demonstrate that a febrile-like temperature enhances mvRNA synthesis and activation of the RIG-I–dependent innate immune response. We also find that activation of RIG-I is dependent on the mvRNA sequence and mvRNA transcription, in line with recent results showing that IAV transcription contributes to innate immune activation (*15*). When we compare pandemic and adapted IAV RNA polymerases, we find that the transcription of mvRNAs by a pandemic IAV RNA polymerase is significantly increased at 39°C. Last, we performed infection experiments to show that the temperature-dependent modulation of the innate immune response is preserved across infections with both H1N1 and H3N2 IAV strains. Overall, we speculate that, depending on the IAV strain and base level of mvRNA synthesis, the activation of the febrile response could create a positive feedback loop that could contribute to improved viral clearance or dysregulation of the immune system.

## RESULTS

### Modeling of influenza virus replication and transcription dynamics

mvRNA and full-length vRNA segment replication and transcription likely occur simultaneously as an IAV infection progresses. Since these processes may affect each other and the accumulation of viral proteins, innate immune signaling, and the biochemical activity of the viral replication complex, it is hard to intuit how changes in this network of viral and host interactions affect the outcome of an infection. To better understand how a change in temperature impacts the dynamics of this network, we developed a mathematical model for a single IAV replication cycle. Building on previous IAV infection models (*24*), we account for nuclear export, the formation of mvRNAs, activation of RIG-I, and explicitly define IAV cap-snatching of host (pre-)mRNAs caps as a limiting resource [and thus that other resources such as nucleotide triphosphates (NTP), lipids, and amino acids are in excess]. In addition, we incorporated recent biochemical and structural findings, including that replication and transcription occur in a complex consisting of the vRNA polymerase and host factor acidic leucine-rich nuclear phosphoprotein 32 kDa (ANP32) (we define this as a single complex Sv) (*25*); that NP is required for full-length segment replication, but not mvRNAs; and that mvRNAs can be replicated and transcribed (Fig. 1B) (*14*). In addition, we assumed that mvRNAs and their transcription products (particularly ccRNAs) can be exported from the nucleus and bind RIG-I to activate an innate immune response (*15*), that the replication of the eight IAV segments (producing vRNA and cRNA) is not fundamentally different, and that expression differences among the segments are driven by variations in transcription (mRNA) and/or translation efficiency. Last, we simplified the existing viral infection model by accounting for only three protein complexes: (i) the vRNA polymerase subunit PA bound to PB1, PB2, and ANP32; (ii) the viral NP; and (iii) the viral nuclear export protein (NEP) bound to M1 and CRM1. For simplicity and to ensure that experimentally testing the model remained feasible, we assumed that other viral proteins—including PA-X, PB1-F2, PB1-N40, NS1, HA, NA, and M2—were not altered by the change in temperature. We parametrized the model using previously published parameters and added biophysical estimates for the added processes [table S1 and (*26*–*31*)]. The resulting model reasonably predicts the abundance of the canonical vRNA species during a single infection cycle in comparison to experimental data (fig. S1, A to D) (*32*).

### Modeling predicts that an increase in temperature lowers NP expression and increases mvRNA expression and innate immune signaling

An increase in temperature above physiological conditions reduces the binding affinities of protein-ligand interactions and the catalytic function of enzymes. Previous experimental results have shown that the binding affinity of the IAV RNA polymerase to RNA is indeed reduced at higher temperatures (*21*). However, simulation of IAV RNA synthesis with reduced binding affinities showed that this change does not inherently increase the rate of mvRNA formation (fig. S1, E and F). Instead, simulating IAV replication at 37° and 39°C predicts that NP levels become reduced from ~2 hours post infection (hpi) at 39°C, while the polymerase levels become elevated from ~5 hpi (Fig. 1C). Decreasing the NP level results in reduced processivity or a failure to encapsidate full-length viral segments into RNPs, and our model predicts that mvRNAs levels at 39°C start to outpace mvRNAs levels at 37°C from 5 hpi onward at the cost of full-length RNA synthesis (Fig. 1, D and E). The imbalance between NP and the vRNA polymerase also accelerates the accumulation and transcription of mvRNAs in the cell (fig. S1G) and the potential for the formation of duplex mvRNA-ccRNAs, which contain both a triphosphate and cap, and their activation of RIG-I (Fig. 1B). Thus, an imbalance in the NP to vRNA polymerase ratio (NP:polymerase < 7) would affect RIG-I activation (Fig. 1F). The effect of this dynamic can be shown experimentally when we titrate NP in a mini-genome assay using a 246-nt-long template from the NP-encoding segment (NP246): As NP availability to the RNA polymerase decreases, full-length template RNA levels decrease, while mvRNA levels and IFN-β promoter activity increase (Fig. 1G), in line with previous observations (*14*). Based on these findings, we propose that the activation of RIG-I is increased at 39°C and particularly sensitive to decreased nuclear protein expression at this temperature.

### Temperature elevation up-regulates the expression of innate immune genes during IAV infection

**Fig. 2.**
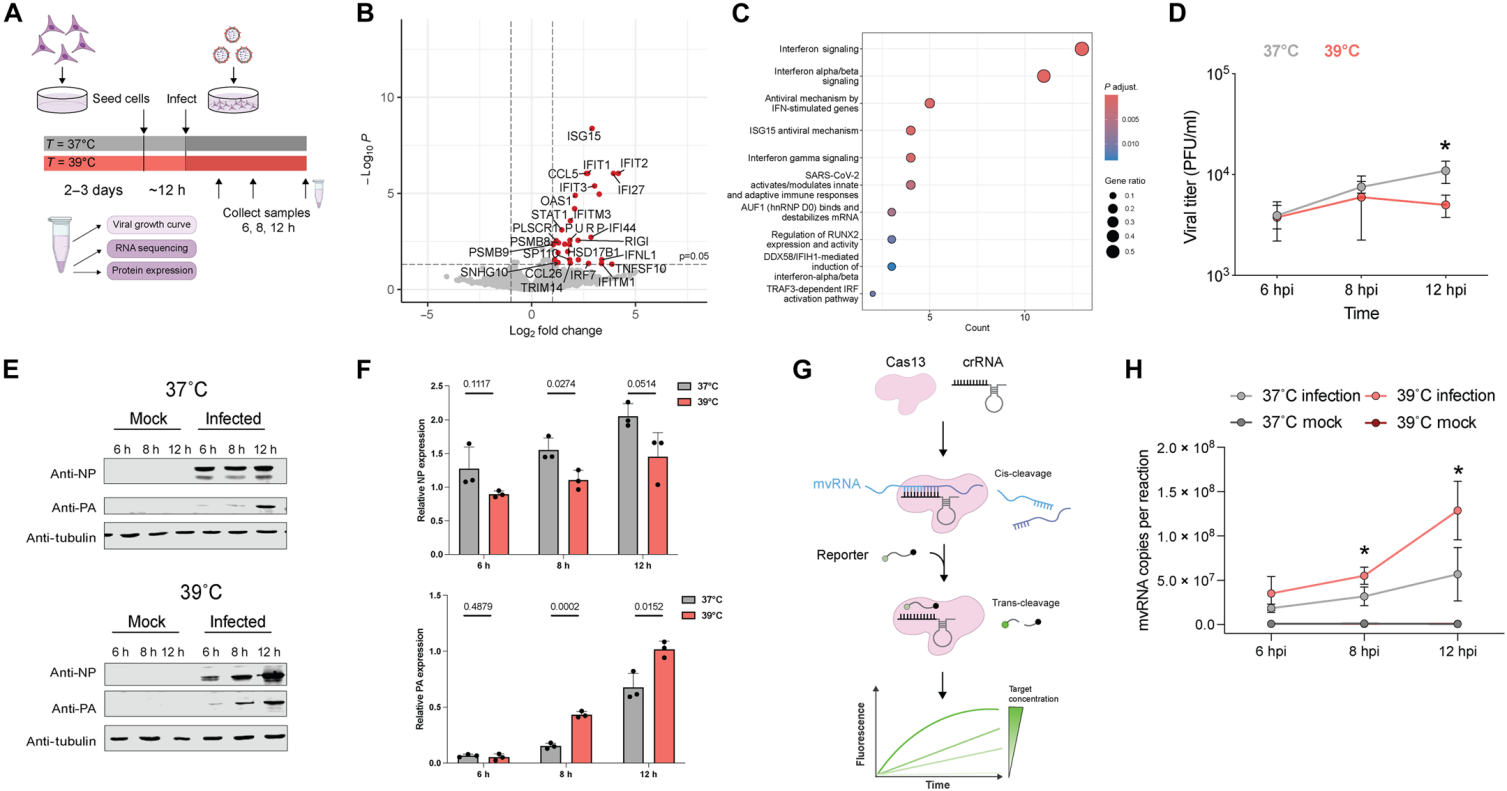
Elevated temperature affects both the innate immune response and aberrant RNA synthesis during infection. (**A**) Schematic of experimental design used for viral infection of A549 cells in cells acclimated at 37° and 39°C. (**B**) Volcano plot illustrating DEGs in A549 cells infected with A/WSN/33 virus at an MOI of 3. RNA-seq was performed on three biological replicates. (**C**) Reactome pathway enrichment analysis of the DEGs. (**D**) Growth kinetics of lab adapted WSN virus in A549 cells at different temperatures (37° or 39°C). A549 cells were infected at an MOI of 3 at 37° and 39°C. The supernatants of the infected cells were harvested at the indicated times, and the virus titers were determined by performing plaque assays in MDCK cells at 37°C. (**E** and **F**) Viral protein and cellular Tubulin expression as analyzed using Western blot. The bar graph depicts the quantified expression level normalized to Tubulin. (**G**) Schematic of RNA detection using Cas13. (**H**) Detection of NP-61 mvRNA using Cas13 assay in fractionated A549 cells infected with WSN. Graph shows the copy number of mvRNA NP-61 in infected cells at 37° and 39°C. Data are shown as a mean of three independent experiments. Error bars indicate SD. The *P* values were determined by using an unpaired *t* test (*P < 0.05).

To confirm the predictions of our model experimentally, we performed A/WSN/33 H1N1 (WSN) infections at 37° or 39°C in temperature-acclimated A549 cells at a multiplicity of infection (MOI) of 3. We extracted total RNA at 12 hpi, quantified host and viral gene expression using RNA sequencing (RNA-seq) (Fig. 2A), and used edgeR to identify differentially expressed genes (DEGs). The only DEGs between the mock infected cells acclimated to 37° or 39°C were three noninnate immune genes (fig. S2A). We observed a comparable result in temperature-acclimated human embryonic kidney (HEK) 293T cells, in which only a few noninnate immune genes were DEGs (fig. S2B and data S1). However, in the infected A549 cells, we found that 31 genes were differentially up-regulated at 39°C relative to 37°C (Fig. 2B). The DEGs included known innate immune signaling factors ISG1, RIG-I, and IRF7 (Fig. 2B). A reactome pathway enrichment analysis confirmed that DEGs at 39°C compared to 37°C were notably enriched in type I IFN, antiviral defense, innate immunity, and ISG15 signaling pathways [*P* < 0.05, false discovery rate (FDR) < 0.05] (Fig. 2C). Together, these results indicate that IAV infection at a simulated febrile temperature causes an up-regulation in antiviral gene expression. To determine whether the incubation temperature influenced the proportion of productively infected cells, A549 cells acclimated to either 37° or 39°C were infected with WSN virus and subsequently analyzed via flow cytometry. Quantitative fluorescence-activated cell sorting (FACS) assessment demonstrated no statistically significant difference in infection rates between the two temperature conditions, indicating that temperature variation within this range does not affect cellular susceptibility to infection (fig. S2, C and D). These findings are consistent with previous reports indicating no significant variation in hemaggluitin protein expression at these temperatures during IAV infection (*33*).

### Temperature elevation increases synthesis of noncanonical RNA products

The transcriptomics data showed a differential response to the IAV infection at 39°C that was independent of the effect of temperature on the transcriptional landscape of the cell (Fig. 2, B and C). To understand how IAV increases innate immune activation in a temperature-dependent manner, we first measured virus growth. As shown in Fig. 2D and fig. S3 (A and B), we observed that IAV titers were modestly but significantly reduced at 39°C relative to 37°C in both A549 and HEK293T cell lines, in line with other reports (*33*). Western blot analysis ruled out that heat shock protein 70 (HSP70), which is known to inhibit influenza virus replication, was responsible for the lower titer levels observed at 39°C as no significant differences in HSP70 expression were observed between 39° and 37°C (fig. S3C). This result suggests that HSP70 likely does not play a direct role in the temperature-dependent inhibition of viral replication, consistent with findings reported by another group (*21*). The analysis of the viral PA and NP levels showed a reduction in NP expression and an increase in PA expression at 39°C, both in a high and low MOI infection setting (Fig. 2, E and F, and fig. S3D), suggesting that either viral mRNA levels or viral protein synthesis was differentially affected for these two viral genes at this temperature. These results were in line with the effect of temperature predicted by our model (Fig. 1).

We next investigated if mvRNA synthesis was increased during infection at 39°C relative to 37°C. Using an amplification-free CRISPR-Cas13 assay that targets the unique mvRNA junction sequence (Fig. 2G), thereby distinguishing mvRNAs from full-length NP-encoding vRNAs, we quantified a 61-nt-long NP-encoding segment-derived mvRNA (NP-61). This mvRNA is highly abundant in ferret lung tissue infected with A/Brevig Mission/1/1918 (H1N1) or A/Indonesia/2005 (H5N1) and A549 cells infected with WSN (*14*, *34*, *35*). As shown in Fig. 2H, we observed a significant increase in the copy number of the NP-61 mvRNA in cells infected with IAV at 39°C relative to 37°C. These results suggest that mvRNAs are produced at higher levels at 39°C compared to 37°C.

### Temperature influences NP and polymerase proteins in distinct ways during an IAV infection

**Fig. 3.**
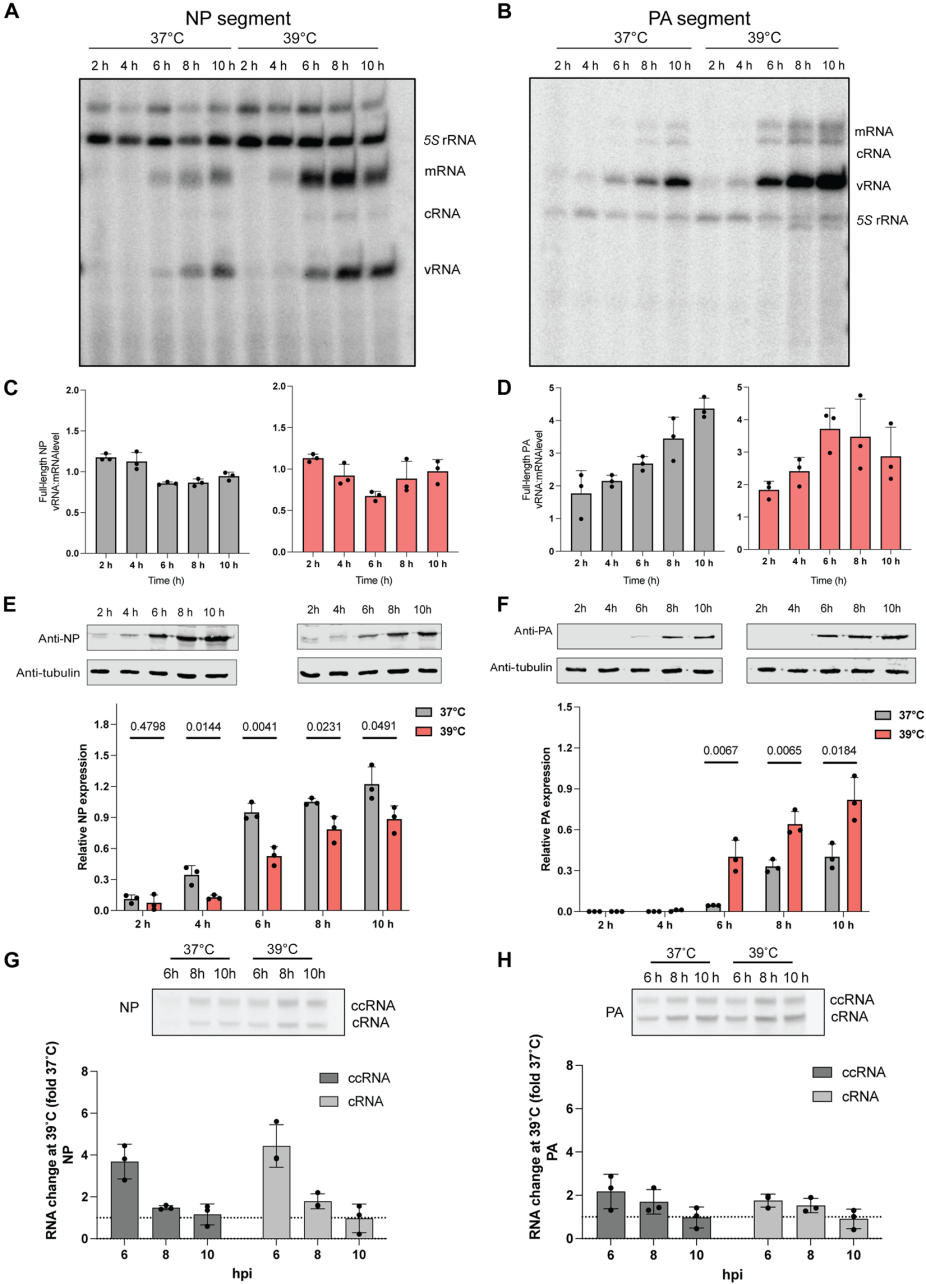
Differential NP and polymerase protein expression at elevated temperature during infection. (**A** and **B**) Steady state mRNA, vRNA, cRNA and 5S rRNA levels as analyzed by primer extension. A549 cells acclimated at 37° and 39°C were infected with an MOI of 1 of A/WSN/33 virus, and RNA samples were taken at different time points post-infection. (**C** and **D**) Normalization of vRNA levels relative to mRNA levels for the NP and PA full length segments. (**E** and **F**) Cell lysates were collected at different time points and analyzed by immunoblotting. Bottom panel shows the quantified Western blot data. (**G** and **H**) Detection of ccRNA and cRNA by TSO-based RT-PCR. Quantification of fold change difference in ccRNA and cRNA levels produced at 39°C versus 37°C. Data are shown as the mean of three independent experiments. Error bars indicate the SD. The *P* values were determined by using an unpaired *t* test. rRNA, ribosomal RNA.

To further validate the predictions of our model and investigate the mechanism underlying the differential NP and PA expression, we infected A549 cells at an MOI of 1 and examined vRNA and NP and PA protein levels (Fig. 3). Quantification of the segment 3 and 5 vRNA and capped RNA (mRNA and ccRNA) levels by primer extension revealed an increase over the course of the infection, with a more pronounced increase at 39°C relative to 37°C (Fig. 3, A to D, and fig. S4, A to D). Western blot analysis (Fig. 3, E and F) showed opposite trends for the NP and PA protein levels: NP was more highly expressed at 37°C compared to 39°C, while the PA RNA polymerase subunit was more highly expressed at 39°C. These findings align with our MOI of 3 experiments (Fig. 2), and they are in line with the decrease in NP expression previously observed in cells infected with IAV and IBV when incubated at 39°C (*33*).

Our results consistently indicate that NP-encoding mRNA levels are high, but that NP protein levels are reduced at 39°C [note that this is below the melting temperature of NP, which is ~45°C (*36*)]. We hypothesized that this discrepancy between the IAV mRNA and protein level might be due to noncanonical transcription termination, which can result in the formation of ccRNAs. In primer extension experiments (fig. S4, C and D), ccRNA and mRNA molecules are indistinguishable from each other because they have the same 3′ terminal sequence (Fig. 1A). However, by adding a template-switch step during reverse transcription that adds a known sequence to the 5′ end of cDNA molecules produced from cRNAs and ccRNAs, the 8- to 14-nt-long primer at the 5′ end of ccRNAs can be used to distinguish ccRNAs from cRNAs by reverse transcription polymerase chain reaction (RT-PCR) (*16*) . Following mRNA depletion and RT-PCR detection, we found an ~4-fold increase in the NP-encoding segment ccRNA levels at 39°C compared to 37°C early in infection, while for PA-encoding segment, we observed only a twofold increase in the ccRNA level at 39°C. Together, these data suggest that segment 5 transcription is more strongly affected by noncanonical transcription termination than segment 3 transcription (Fig. 3, G and H) and that the ccRNA:cRNA ratio for segment 5 is almost twofold higher than segment 3. Although it is currently unclear whether ccRNAs can be translated into protein, we propose that the observed increase in ccRNA levels might contribute to the decreased NP protein expression at 39°C.

### Temperature affects mvRNA replication and transcription in a t-loop–dependent manner

**Fig. 4.**
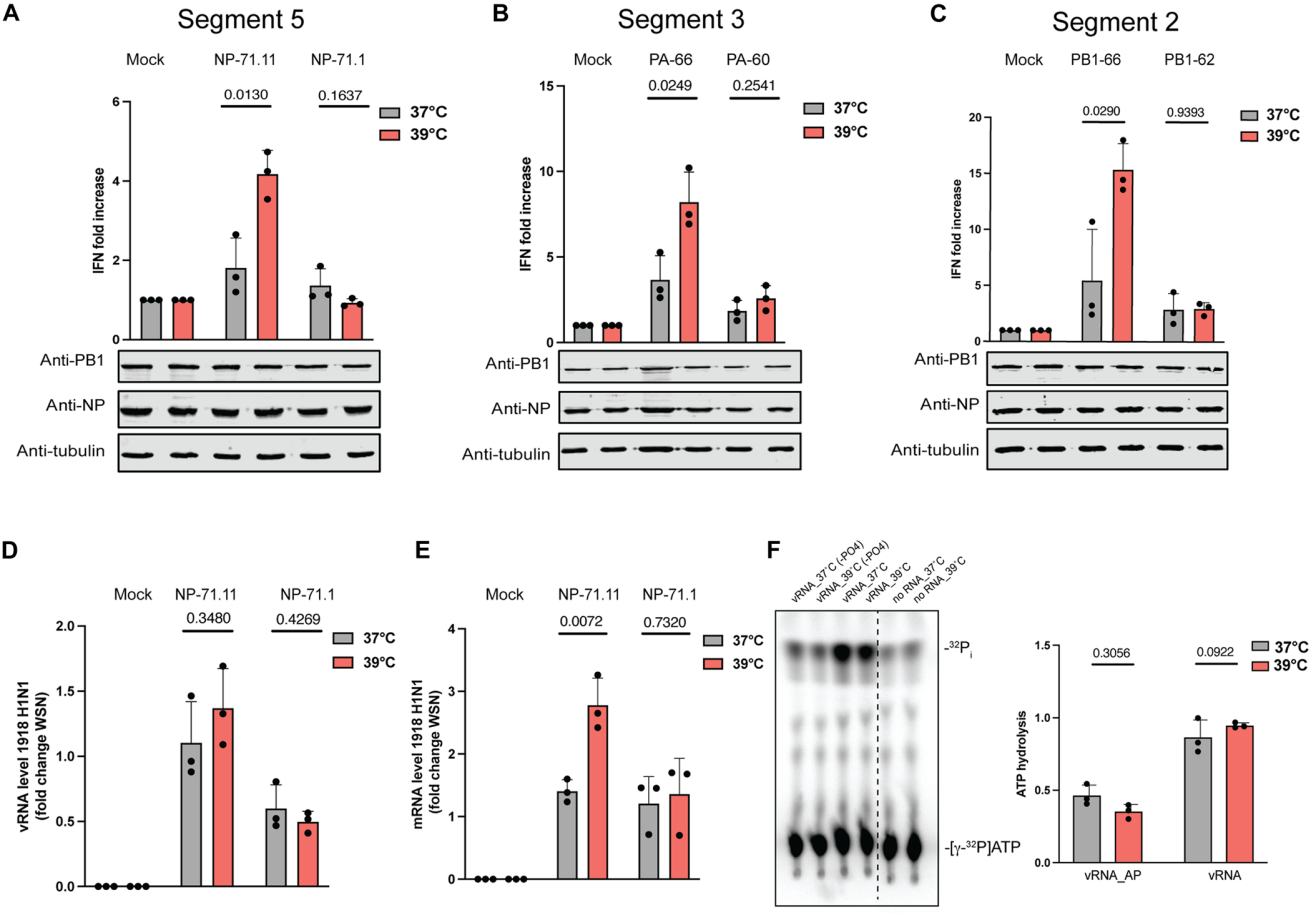
mvRNAs induce increased innate immune signaling at higher temperature. (**A** to **C**) Analysis of IFN-β promoter activity induced by the replication of segment 5, segment 3, or segment 2 mvRNAs 24 hours posttransfection of BM18 IAV RNA polymerase. PB1, NP, and tubulin expression was analyzed by Western blot. (**D**) Normalized vRNA levels for BM18 polymerase relative to WSN polymerase. (**E**) Normalized mRNA levels for BM18 polymerase relative to WSN polymerase. (**F**) ATPase activity of recombinant RIG-I was assessed in the presence of in vitro transcribed PA66 mvRNA, dephosphorylated PA66 mvRNA, or a no RNA control. Data are shown as the mean of three independent experiments. Error bars indicate SD. *P* values were determined using a two-sided, unpaired *t* test.

mvRNAs are diverse in sequence (*35*), but only mvRNAs that contain a t-loop can activate RIG-I (*15*). We previously showed that the RNA polymerases from both human and avian-adapted IAV strains are sensitive to t-loops. mvRNAs can only be studied using transfection experiments because currently no tools exist to manipulate individual mvRNAs during infection. To confirm that the t-loop–containing mvRNAs also triggered higher IFN-β reporter activities at 39°C, we transfected the IAV RNA polymerases from WSN or A/Brevig Mission/1/18 H1N1 (BM18) alongside an IFN-β reporter and one of six mvRNAs derived from three different segments: PA-60 and PA-66, which are derived from segment 3; NP-71.11 and NP-71.1, which are derived from segment 5; and PB1-62 and PB1-66, which are derived from segment 2. For the transfection, we used 39°C-acclimated HEK293T cells, which show no difference in innate immune signaling compared to cells acclimated to 37°C (fig. S2B). Luciferase signals were measured 24 hours after transfection and were normalized to the cotransfected *Renilla* luciferase transfection control. Our mini genome assay revealed that mvRNAs that contained a t-loop (NP-71.11, PA-66, and PB1-66) induced a significantly higher IFN-β reporter activity at 39°C than 37°C (Fig. 4, A to C, and fig. S5, A to C). In contrast, the IFN-β reporter activity between the two temperatures was not different for mvRNAs that did not contain a t-loop (NP-71.1, PA-60, and PB1-62) (Fig. 4, A to C, and fig. S5, A to C). We observed these differences in IFN-β reporter activity for both the WSN and BM18 RNA polymerases. As a control, we compared the IFN-β reporter activity induced by the replication of full-length segments 2, 3, and 5 and observed no significant difference between the two temperatures (fig. S9A). Together, these results indicate that an increased activation of the innate immune response at 39°C is dependent on how transient RNA structures modulate IAV RNA polymerase activity on mvRNAs.

We previously showed that t-loops affect IAV RNA polymerase processivity (*35*). To investigate whether the increased IFN-β reporter activity was caused by a further decrease in RNA polymerase activity, we extracted RNA from the mini-genome assay and measured vRNA levels using primer extension analysis. For the BM18 RNA polymerase, two of the mvRNAs with a t-loop (NP-71.11 and PA-66) showed reduced vRNA steady-state levels at 39°C compared to 37°C (fig. S6, A and B), while the third with a t-loop, PB1-66, did not show a significant reduction in vRNA steady-state level between the two temperatures (fig. S6C). The three mvRNAs without a t-loop (NP-71.1, PA-60, and PB1-62) showed no difference in the vRNA steady-state levels between the two temperatures (fig. S6, A to C). For the WSN RNA polymerase, we observed reduced replication steady-state levels for all three mvRNAs with a t-loop and no differences for the three mvRNAs without a t-loop (fig. S7, A to C).

For the mRNA steady-state levels produced by the BM18 RNA polymerase, we found no significant differences for all six mvRNA templates, with or without the t-loop, between 37° and 39°C (fig. S6, D to F). However, in the presence of the WSN polymerase, mvRNAs NP-71.11 and PA-66 showed a notable decrease in mRNA levels, while the other four templates showed no changes (fig. S7, D to F). Last, we measured the production of ccRNA molecules and observed a significant decrease for NP-71.11 and PA-66 templates with both the BM18 H1N1 and WSN polymerases at 39°C compared to 37°C. For the other four mvRNA templates, we observed no reduction in ccRNA level (fig. S6, G to I, and fig. S7, G to I). Given that there were slight differences between the steady-state RNA levels produced by the BM18 and WSN RNA polymerase, we compared the relative vRNA and mRNA levels produced on mvRNA templates NP71.11 and NP71.1. As shown in Fig. 4 (D and E), we found that although the replication levels remained consistent between the two polymerases, there was a significant increase in t-loop containing mvRNA transcription by the BM18 RNA polymerase relative to the WSN enzyme at 39°C.

To confirm that IAV transcription initiation is affected by the increase in temperature, we measured cap-snatching on model vRNA and cRNA promoters and 71-nt-long mvRNA derived from segment 5 (NP71.1). To this end, we first purified the WSN RNA polymerase and incubated it with a radiolabeled, capped 20-nt-long RNA in the presence of model vRNA and cRNA promoters or NP71.1 in their positive or negative sense at 30°, 37°, and 39°C. As shown in fig. S8A, the endonuclease activity was minimal when no template was added to the reaction, while it was the most efficient when the vRNA promoter or a vRNA-sense mvRNA was present. The highest endonuclease activity was observed at 30°C. We also observed cap cleavage activity in the presence of the cRNA promoter and the positive-sense mvRNA templates.

We next measured if transcription initiation was influenced by temperature. To this end, we incubated the purified IAV polymerase with a radiolabeled 11-nt-long capped primer ending in 3′ AG or 3′ CA and either the wild-type vRNA promoter or mutant 3′ U1A promoter at 30°, 37°, and 39°C (fig. S8B). In this assay, the vRNA polymerase aligns the capped primer to the terminal sequence of the template, and mismatched base pair reduced the efficiency of transcription initiation. As shown in fig. S8B, we observed a temperature-dependent reduction in transcription initiation but noted that initiation after base pairing with nucleotide 2 of the primer was more temperature-independent in two of the three primer-template combinations. Together, these results are mostly in line with the reduced mRNA levels produced from the mvRNA templates we observe in our mini-genome assays (Fig. 4). The results contrast the higher capped RNA levels produced from the full-length vRNA templates at 39°C during infection (Fig. 3), suggesting that the transcription efficiency of short mvRNA-like templates is different from NP-bound full-length RNA molecules. Presently, the underlying mechanism is not understood.

### Temperature dependent response to mvRNAs is RIG-I dependent

To confirm that the activation of innate immune signaling by mvRNAs at 39°C was RIG-I dependent, we used a HEK293 *RIG-I*^−/−^ cell line. The knockout cells were transfected with mvRNA templates NP-71.11 and PB1-66, which both induce IFN-β promoter activity, and mvRNA templates NP-71.1 and PB1-62, which do not induce IFN-β promoter activity. In all transfections, we were unable to observe IFN-β promoter induction in the knockout cell line at 39°C, thus implying that the induction of IFN-β promoter activation by mvRNAs is RIG-I dependent at both 37°C and 39°C (Fig. S9B-C).

Finally, to confirm that the difference in IFN-β promoter activation at 39°C was not triggered by a higher activity of RIG-I, we performed an adenosine triphosphatase (ATPase)–hydrolysis assay using recombinant RIG-I. The ATPase activity of RIG-I was assessed in the presence of in vitro transcribed PA-66–based vRNA and cRNA at 37° and 39°C. As negative controls, we also used alkaline phosphatase–treated vRNA and cRNA transcripts. As shown in Fig. 4F, no significant difference was observed in the RIG-I activity at the two temperatures (Fig. 4F and fig. S9D), which implies that the RNA binding–dependent adenosine 5′-triphosphate (ATP) hydrolysis activity of RIG-I likely did not contribute to the observed increase IFN-β promoter activation at 39°C.

### Innate immune modulation by temperature is conserved across infections with H1N1 and H3N2 IAV strains

**Fig. 5.**
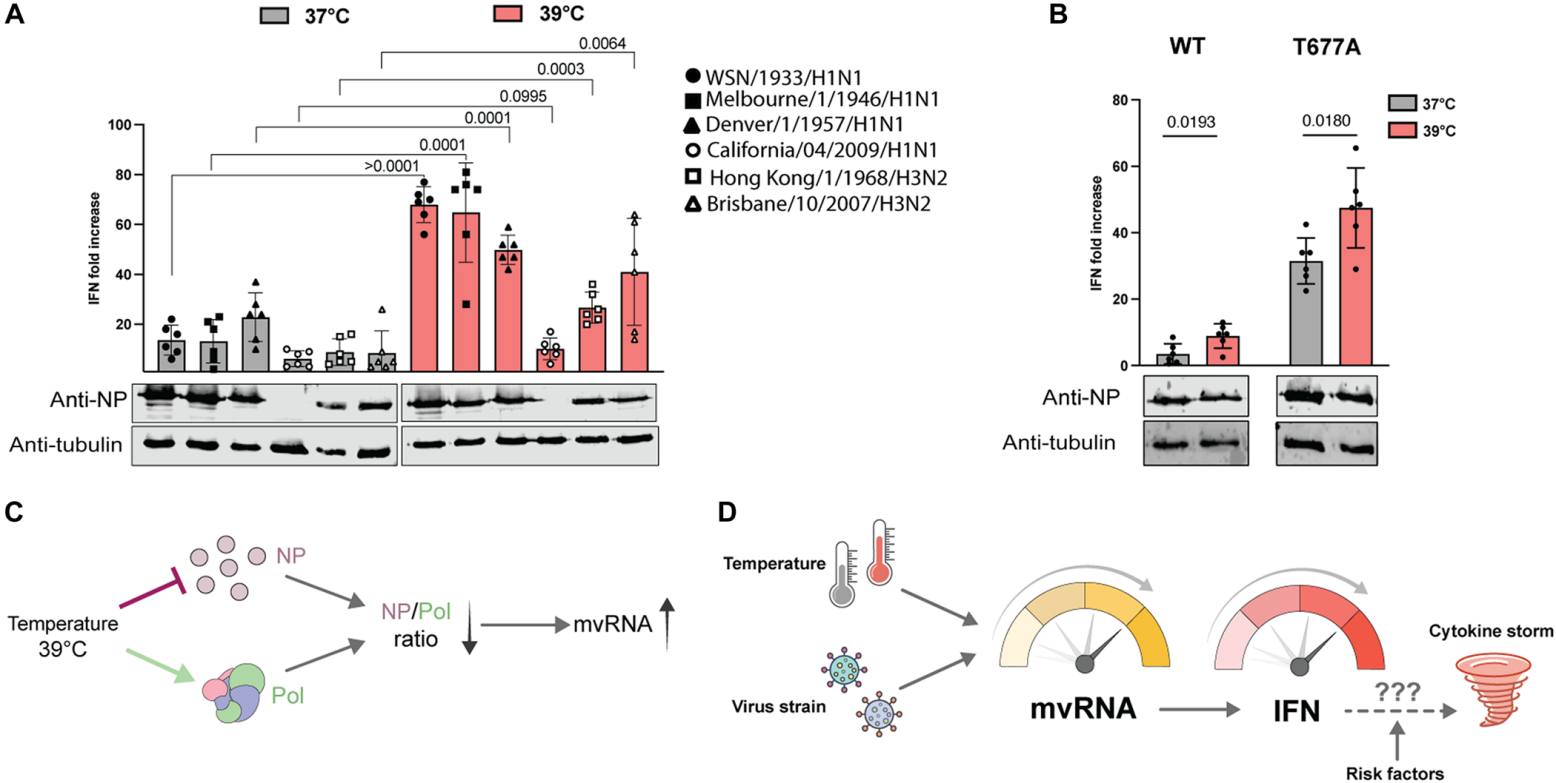
Temperature-driven modulation of innate immunity aligns with responses to other IAV strains. (**A**) Analysis of IFN-β promoter activity, 12 hpi, in HEK-luc cell line adapted at 37° and 39°C, infected with WSN, Melbourne46, Denver57, Cal09, HK68, and Brisbane07 IAV strains at an MOI of 3. The NP and tubulin expression was analyzed by Western blot. (**B**) IFN-β promoter activity was assessed in HEK-luc cells 12 hpi. The cells were adapted to 37° and 39°C and infected at an MOI of 1 with either the WSN strain or the T677A mutant virus strain. The NP and tubulin expression was analyzed by Western blot. Data are shown as the mean of six independent experiments. Error bars indicate SD. *P* values were determined using a two-sided, unpaired *t* test. (**C**) Increase in infection temperature can differently affect polymerase and NP expression, and this imbalance may contribute to the synthesis of mvRNAs. (**D**) Formation of mvRNAs is influenced by both temperature and the viral strain infecting the host, which can affect activation of the innate immune response, and cytokine and chemokine expression. A fever start may create a positive feedback loop that leads to increased mvRNA production and innate immune activation and which may ultimately contribute to a cytokine storm. The likelihood for a cytokine storm may be modulated by risk and other host and viral factors.

To confirm that temperature influences innate immune activation, we conducted infections using a panel of five additional influenza virus strains in HEK-luc cells: A/Melbourne/1946 (H1N1), A/Denver/1957 (H1N1), A/California/07/2009 (H1N1), A/Hong Kong/1968 (H3N2), and A/Brisbane/2007 (H3N2). All strains, with the exception of A/California/07/2009 (Cal09), exhibited a significantly elevated innate immune response at 39°C relative to 37°C, indicating a temperature-dependent enhancement of immune activation (Fig. 5A). The expression levels of viral proteins from Cal09 were markedly low, suggesting limited viral infection or replication capacity in our HEK-luc cells and explaining the minimal activation of the innate immune response observed.

Our model and previously published data indicate that both mvRNAs and ccRNAs are needed for innate immune activation and that both are differentially expressed at 39°C. As a further validation for our model, we performed infections with a recombinant WSN virus containing a T677A substitution in PB1 (*16*). This mutation results in increased ccRNA levels and innate immune activation relative to wild-type WSN without increasing mvRNA levels. If our model were correct, then elevated ccRNA starting levels would push the innate immune response further at 39°C. As shown in Fig. 5B, infection with WSN PB1 T677A led to higher elevated IFN-β reporter activity at 37°C relative to the wild-type strain, in line with our previous observations (*16*). Moreover, and as predicted, at 39°C, the innate immune induction was further amplified, highlighting a temperature-dependent enhancement of the mutant’s immunostimulatory potential relative to the wild-type virus (Fig. 5, C and D)

## DISCUSSION

Despite its impact on human morbidity, the fever response remains one of the least understood aspects of an acute inflammatory reaction to infection. We here explored the impact of this increase in temperature on IAV noncanonical RNA synthesis and the innate immune response against IAV infection in vitro. We find that IAV RNA synthesis on full-length segments is not reduced at 39°C, but we do observe increased levels of segment 5 ccRNA synthesis and a corresponding decrease in NP levels. The reduced NP levels lead to an imbalance between the vRNA polymerase and NP, which increases mvRNA synthesis and IFN-β promoter activation (Figs. 2 and 4). These dynamics can be captured by a relatively simple model of IAV infection (Fig. 1).

It is tempting to speculate that these dynamics could lead to a positive feedback loop during infection with a human or avian IAV. Following IAV infection and the initial production of mvRNAs, the innate immune response is activated and a moderate-grade fever is triggered (e.g., 39°C). At this elevated temperature, the IAV RNA polymerase starts to produce additional mvRNAs, which sustain the activated innate immune and febrile response. Such an immune response may lead to improved viral clearance in line with studies of respiratory virus infections (*37*–*39*). However, the relation between innate immune activation and disease outcome is complex, highly variable, and still poorly understood (*40*–*42*). Moreover, immune cells show altered immune gene expression in response to febrile temperatures, even in the absence of infection (*43*, *44*).

It is possible that a prolonged innate immune response blocks the activation of negative feedback loops and/or contributes to a dysregulation of the innate immune response, which is a hallmark of infections with highly pathogenic influenza virus strains (Fig. 5C). This scenario would be particularly likely for infections with IAV strains that produce higher mvRNA base levels, such as the pandemic 1918 H1N1 IAV (*14*). At a higher base mvRNA level, the impact of fever would rapidly escalate mvRNA production and innate immune activation (Fig. 5D), potentially contributing to the dysregulated innate immune response observed in highly pathogenic avian and pandemic IAV infections (*3*, *45*). All the above effects and outcomes are likely modulated by immune modulating viral proteins—such as PA-X, NS1, and PB1-F2—and the host adaptive immune response and the microbiome (*46*–*50*).

Temperature is a critical factor in influenza virus infection and broadly influences the molecular machinery of respiratory RNA viruses across hosts and during spillover events. Viruses from birds, bats, and cold-blooded animals replicate at higher or ambient temperatures, while human-adapted viruses such as IAV, coronaviruses, respiratory syncytial virus, and rhinovirus are optimized for the cooler conditions of the upper respiratory tract (32° to 33°C) (*10*). Spillover viruses often retain replication preferences suited to their reservoir hosts body temperatures, which may hinder their adaptation to the human respiratory environment. A key component in this adaptation process is the RNA polymerase, which is sensitive to temperature and may influence the production of aberrant vRNAs (*51*, *52*). Our study highlights strain-specific differences in innate immune responses, which are consistently elevated at 39°C and may be influenced by differences in mvRNA production. Human-adapted strains replicate efficiently at upper respiratory tract temperatures with minimal innate immune activation, raising the question whether mvRNAs are produced or even suppressed under these conditions. Future studies could also investigate how IAV strains from avian and bat hosts respond to febrile temperatures, particularly in relation to aberrant RNA synthesis and immune activation.

Overall, our study incorporates cell culture–based assays, in vitro assays, and mathematical modeling and offers a comprehensive dynamic perspective into the temperature-dependent effects on viral molecular machinery. In addition, the findings of this study underline the role of mvRNAs in innate immune activation and reveal that multiple rounds of aberrant RNA synthesis at febrile temperatures can stimulate innate immune activation.

## MATERIALS AND METHODS

### Model for influenza replication with noncanonical RNA products

The model of the IAV replication dynamics was constructed by identifying the essential steps to capture replication and protein expression dynamics from a previously published model of intracellular IAV replication dynamics (*24*). The steps include cRNA production from nuclear RNPs, vRNA production from cRNA, RNP assembly from polymerase, nucleoprotein, and c- or vRNA, transcription of nuclear proteins making mRNA, translation of mRNA into protein, degradation of RNA, protein and RNP, and export of RNPs from the nucleus, gated by NEP. We then extend this model by three processes described in recent literature: (i) Transcription of mRNA influenza requires stealing CAP sequences produced by the host (CAP-snatching); (ii) replication requires a second polymerase complex to bind an RNP, and replication is only completed when the second complex binds to nucleoprotein; otherwise, (iii) incomplete replication leads to the formation of mvRNA. These products replicate to form positive and negative sense RNA products (mini vRNA or cRNA) and transcribe into capped aberrant mRNA products. On the other hand, we simplify the binding kinetics by assuming all complex formations follow the same diffusion-limited rate constant and are only distinguished by binding affinities (table S1) (*53*). From these assumptions, we formulate a differential equation model that describes the time evolution of the 23 species (table S2). Except for CAP-snatching and RNP formation during replication, all processes are modeled using mass action kinetics. New RNP formation from replication follows a Michaelis-Menten like kinetics of the form

$$v_{r,i}=k_{r,i}\;\left[{\mathrm{RNP}}_{i},\text{Pol},\text{NP}\right]\frac{\left[\text{NP}\right]}{\left[\text{NP}\right]+K_{\text{Pol},\text{NP}}}$$

where i∈[c,v] denotes the RNA type, $k_{r,i}$ is the respective replication rate, $\left[\text{RN}P_{i},\text{Pol},\text{NP}\right]$ is the number of c or vRNA RNP polymerase nucleoprotein complexes, [NP] is the number of NPs, and $K_{\text{NP},\text{Pol}}$ is the binding affinity of nucleoprotein to the polymerase complex. CAP-snatching kinetics are modeled similarly

$$v_{t,j}=k_{t,j}\;\left[{\mathrm{RNP}}_{i},\text{Pol}\right]\frac{\left[\text{CAP}\right]}{\left[\text{CAP}\right]+K_{\text{Pol},\text{Cap}}}$$

where $v_{t,j}$ with j∈[np,pol,nep] denotes the transcription rate for mRNA encoding nuclear protein, polymerase protein, and nuclear exit protein, respectively; $\left[\text{RN}P_{i},\text{Pol}\right]$ is the number of c or vRNA RNP polymerase complexes; [CAP] is the size of the CAP-pool; and $K_{\text{Pol},\text{Cap}}$ is the binding affinity of the polymerase complexes to the CAP sequence.

The model was parameterized by directly or indirectly incorporating the parameter values identified in previous modeling efforts (*24*) wherever possible. In the next step, all binding affinities were parameterized using data from in vitro assays. This left us with three undetermined parameters: The effective replication volume $V_{r}$, the CAP synthesis rate $V_{\text{cap}}$, and the CAP degradation rate $k_{d,\text{cap}}$. We found that volume $V_{r}$ needs to be a fraction (~1%) of the nuclear volume for transcription to begin within 1 to 2 hours after infection. Considering that a single RNP only interacts with a small part of the nucleus, we compute an order of magnitude estimate of the CAP synthesis rate: $V_{\text{cap}}$ in the vicinity of the RNP is about 1% of the total mRNA synthesis rate, i.e., thousands of mRNA per hour (*54*, *55*)

$$V_{\text{cap}}\approx 0.01\times 100,000\frac{\#\text{mRNA}}{h}\approx 10^{3}\frac{\#\text{CAP}}{h}$$

Last, we set effective cap degradation rate $k_{d,\text{cap}}$ to match experimentally observed mRNA, cRNA, and vRNa dynamics (fig. S1, B to D) (*32*). We recognized that the TPM values reported (transcript per kilobase per million) approximately match the RNA per cell quantified by RT-PCR (*56*) or IAV infection of Madin-Darby canine kidney (MDCK) cells and that RNA levels for infections in A549 cells are about 10-fold lower than in MDCK cells. We find $k_{d,\text{cap}}=0.1$ by matching the 10×-scaled predictions for A549 cells (fig. S1A) to data measured for MDCK infection (fig. S1, B to D). We compute the time evolution of all species by numerically solving the ordering differential equations with initial conditions modeling the presence of a single viral particle and the steady-state level of CAPs before infection, i.e., ${\left[\text{CAP}\right]}_{t\to \infty }=V_{\text{cap}}$/$k_{d,\text{cap}}$. To predict the effect on the innate immune response, we assume that double-stranded minis are the main driver and bind the RIG-I receptor with dissociation constant of 2.5 nM (*26*).

### Cell culture and viruses

HEK cells (HEK293T) cells and alveolar basal epithelial cells (A549) cells were maintained in complete media [Dulbecco’s modified Eagle’s medium (DMEM; Sigma-Aldrich) supplemented with 10% fetal calf serum (FCS; Gibco) and 2 mM l-glutamine (Gibco)] at 5% CO_2_. HEK293T and MDCK cells were sourced from the American Type Culture Collection. Wild-type and *RIG-I*^−/−^ human embryonic kidney cells stably expressing firefly luciferase and green fluorescent protein under the *IFN-b* promoter (HEK293-luc) were a gift from J. Rehwinkel (Oxford University) and have been validated before (*57*). Wild-type A549 were provided by B. Ferguson (Cambridge University) (*58*). A/WSN/1933 (H1N1) and T677AΔPB1 A/WSN/1933 (H1N1) virus were generated in the laboratory. Influenza A/Melbourne/1/1946 (H1N1), Influenza A/Denver/1/57 (H1N1), Influenza A/California/04/2009 (H1N1), Influenza A/Hong Kong/1/68 (H3N2), and Influenza A/Brisbane/10/2007 (H3N2) were ordered from Biodefense and Emerging Infections (BEI) resources. Before all experiments, cells cultured at 37°C were split into independent but paired flasks and incubated at 37° and 39°C for 72 hours. MDCK cells used for plaque assays were incubated at 37°C.

### Plasmids

The pPolI plasmids encoding A/WSN/33 genome segments were previously described (*59*). The pPolI plasmids encoding segment 2–, segment 3– and segment 5–based templates with internal deletions were described in (*14*) and table S3; the pcDNA3 plasmids encoding wild-type A/WSN/33 RNP proteins, the plasmid-expressing firefly luciferase under *IFN-b* promoter (pIFD(−116)lucter), and pcDNA3-encoding *Renilla* luciferase were described in (*14*).

### Virus infections

For infection experiments, A549 cells were seeded in 12-well plates at 2 × 10^5^ cells per well in complete DMEM. The next day, cells were infected with an MOI indicated for specific experiments in infection DMEM at 37°C and 5% CO_2_ for 1 hour. After infection, cells were washed with phosphate-buffered saline (PBS) and incubated in infection DMEM at 37° or 39°C in 5% CO_2_. At the time periods indicated for specific experiments, supernatants were collected for virus titer quantification by plaque assay, and cells were lysed in either TRI reagent (Sigma-Aldrich) for phenol-chloroform RNA extraction and isopropanol precipitation as described previously (*60*) or Laemmli buffer for Western blotting.

### IAV growth kinetics at 37° and 39°C

For single and multicycle IAV infections, A549 cells were seeded and incubated at the two temperatures. Cells were infected with A/WSN/33 at an MOI of 3, 1, or 0.01 plaque-forming units per cell in DMEM for 1 hour. The inoculum was the removed, and cells were further incubated for different time points in growth media at the two temperatures, 37° or 39°C. Supernatants were then titrated on MDCK cells by plaque assay at 37°C.

### RNP reconstitution and IFN-β promoter activity assay

HEK293T cells acclimated at 37° or 39°C for 3 days were transfected in 12- or 24-well plate format with pcDNA3 plasmids (0.25 μg per well) encoding PB2, PB1, PA (wild-type WSN), and NP and pPolI plasmid (0.25 μg per well) encoding a full-length vRNA segment or an internally truncated segment (table S3) under the control of the PolI promoter (*14*). pCAGGS plasmids encoding PB2, PB1, PA, and NP were used in case of 1918 H1N1 reconstitution assay. For assessment of *IFN-*β promoter activity, 0.1 μg of a plasmid expressing firefly luciferase from the *IFN-*β promoter and 0.01 μg of a pCAG plasmid–expressing *Renilla* luciferase driven by the chicken β-actin promoter with a cytomegalovirus enhancer were added to the transfection mix. The transfection was performed using a 1:2.5 DNA (μg)/Lipofectamine 2000 (Invitrogen) ratio. Twenty-four hours later, the DMEM was aspirated out. Next, we used PBS to detach the cells and split into three fractions: RNA isolation (one-half cell pellet), Western blotting (one-fourth cell pellet) or the *IFN-*β promoter activity assay (one-fourth cell pellet). For *IFN-*β promoter activity assays, 25 μl of DualGlo reagent (Promega) was added to 25 μl of cells suspended in PBS. This mixture was then incubated for 10 min, and the firefly luciferase readings were taken using a Synergy LX plate reader (BioTek). Next, 25 μl of Stop-Glo reagent per well was added to each well and incubated for 10 min, and the *Renilla* luciferase readings were taken. For the final analysis, firefly luciferase values were normalized by the *Renilla* luciferase values.

### Cas13 assay

We used the previously established Cas 13 assay to quantify mvRNA in our sample (*34*). The Cas13-based detection reactions contained 10 nM LbuCas13a, 20 mM Hepes (pH 8.0), 60 mM KCl, and 5% polyethylene glycol, ribonuclease (RNase) inhibitor murine (2 U/μl; New England Biolabs), 14 mM MgOAc, 0.25 μM 6UFAM (FAM-UUUUUUU-IowaBlack), 5 nM crRNA (GACCACCCCAAAAAUGAAGGGGACUAAAACAGAUAAUCACUCACAGAGUGACAUCGAA), and the reported amount of target RNA (~50 ng/μl). Each reaction was first mixed in a volume of 44 μl in a 96-well plate. Following mixing, 20 μl of sample was transferred to 384-well plates in duplicate. The plate was then placed in a BioTek Synergy H1 Plate Reader and incubated at 37°C for 3 hours. The fluorescence of each reaction was measured at 5-min intervals. The curves were analyzed as described previously (*34*).

### mvRNA PCR

For the mvRNA RT-PCR in Fig. 1G, we used the method described previously (*14*).

### Western blotting

For protein level analysis, cells were first lysed in Laemmli buffer [62.5 mM tris-HCl (pH 6.8)], 10% glycerol, 2% SDS, 100 mM dithiothreitol (DTT) (Invitrogen), and 0.01% bromophenol blue), followed by a short sonication. Next, proteins were separated using an 8% SDS–polyacrylamide gel electrophoresis (SDS-PAGE) gel, followed by immobilization on a 0.45- or a 0.25-μm nitrocellulose membrane (GE healthcare). Next, the membranes were incubated in blocking buffer {PBS, 5% bovine serum albumin [Research Products International (RPI)], 0.1% Tween 20 (RPI)} for 1 hour. We then incubated the membranes with the primary antibody diluted in blocking buffer overnight at 4°C. Primary antibodies used in the study are described in table S5. The following day, the membranes were washed 3× using PBST (PBS and 0.05% Tween 20). The binding of the primary antibodies was detected using secondary antibodies sourced from LI-COR (table S5). After 2-hour incubation with 1:10,000 dilution of secondary antibodies in blocking buffer at 4°C, membranes were washed 3× with PBST. Next, fluorescent signals were detected using an Odyssey DLx scanner (LI-COR). Image Studio Lite software (LI-COR) was used for quantification.

### Primer extension analysis

Primer extensions were performed essentially as described previously (*60*). Briefly, total RNA was reverse-transcribed using SuperScript III (Invitrogen) and ^32^P-labeled primers targeting the vRNA of interest (vRNA, cRNA, and mRNA) along with the cellular 5*S* ribosomal RNA (rRNA) loading control (table S4) in 10-μl reactions at 50°C for 1 hour. Reactions were stopped using 10 μl of formamide loading dye (90% formamide, 10 mM EDTA, 0.25% bromophenol blue, and 0.25% xylene cyanol FF) and denatured at 95°C for 2 min. cDNA products were resolved on a denaturing acrylamide gel [6 or 12% or 20% acrylamide (19:1, Bio-Rad)], 7 M urea, 1x tris-borate EDTA buffer (2 mM EDTA, 89 mM boric acid, and 89 mM tris), 0.1% tetramethylethylenediamine (TEMED), 0.06% (w/v) ammonium persulfate (APS) in 1× tris-borate EDTA running buffer for 1.5 to 3 hours at 35 W. In case of a 6% gel, the gel had to be dried. Last, the radiolabeled signals were visualized using phosphor imaging plates (FujiFilm) on a Typhoon FLA 9000 scanner (GE Healthcare). Image Studio Lite software (LI-COR) was used for signal quantification. The quantified signal for RNA species was normalized to its corresponding 5*S* rRNA signal.

### IAV RNA polymerase purification

Wild-type IAV RNA polymerase was purified using tandem-affinity purification (TAP) as described previously (*60*) with modifications. Briefly, 4 μg of pcDNA3 plasmids expressing wild-type PB1, PA, and PB2-TAP were transfected into HEK293T cells using 60 μg of polyethylenimine (PEI) in Opti-MEM (Gibco). Forty-eight hours posttransfection, cells were first harvested in PBS, followed by a wash with PBS. Cells were then lysed in 1 ml of lysis buffer [50 mM Hepes (pH 7.5), 200 mM NaCl, 25% glycerol (Sigma-Aldrich), 2% Tween 20 (RPI), 1 mM β-mercaptoethanol (Bio-Rad), and 1x EDTA-free Protease inhibitor cocktail (Roche)] at 4°C for 1 hour. The lysed cells were next sonicated to disrupt DNA and RNA molecules and centrifuged at 17,000*g* for 5 min at 4°C. The cleared lysates were then bound to 50 μl of immunoglobulin G Sepharose beads 6 Flow (GE Healthcare). The beads were prewashed 3× in binding buffer [50 mM Hepes (pH 8), 200 mM NaCl, 25% glycerol, and 2% Tween 20]. The binding was performed under constant rotation at 4°C for 2 hours. Next, the beads were washed 3× with binding buffer and 1× with cleavage buffer [50 mM Hepes (pH 7.5), 200 mM NaCl, 25% glycerol, 0.5% Tween, and 1 mM DTT]. Last, the TAP-tag was removed using Tobacco etch virus protease (Invitrogen, #12575-015) in 250 μl of cleavage buffer. Cleavage was performed at 4°C for approximately 16 hours. The beads were separated from the cleaved RNA polymerase by centrifugation at 500g for 1 min. The partially purified RNA polymerase was next analyzed through SDS-PAGE, and silver staining was performed using a SilverXpress kit (Invitrogen).

### In vitro transcription and RNA transfection

DNA templates for in vitro transcription were prepared by PCR using the pPolI-PA66 plasmid as a template as described previously (*14*). In vitro transcriptions were performed using the MEGAshortscript T7 Transcription Kit (Thermo Fisher Scientific) according to the manufacturer’s protocol. The products were resolved using 15% denaturing PAGE [7 M urea, 1x tris-borate EDTA buffer, 0.1% TEMED, and 0.06% (w/v) APS] in 1× tris-borate EDTA running buffer. The products were gel-purified and desalted using an RNA Clean & Concentrator-5 (Zymo Research). The quality of the final RNA preparations was assessed using denaturing acrylamide gel electrophoresis.

### ATP hydrolysis assay

RIG-I was purified as described previously (*61*). For ATPase assays, 0.5 μM RIG-I was incubated with 0.1 μM [γ-^32^P]ATP (6000 Ci/mmole, Revvity) and 10 ng of in vitro transcribed template RNA as described above. Activity assays were performed in a buffer containing 50 mM Hepes (pH 8.0), 150 mM NaCl, 2 mM MgCl_2_, and 5 mM DTT and quenched using 1 M formic acid/50 mM EDTA. [γ-^32^P]ATP and ^32^P_i_ were resolved using glass-backed PEI-cellulose TLC plates (Sigma-Aldrich) in 0.4 M KH_2_PO_4_ (pH 3.4). After wrapping the TLC plates in plastic foil, the radioactive signals were detected using BAS-MS phosphor imaging plates (FujiFilm) through a 1-hour exposure and visualized using a Typhoon FLA 9000 scanner (GE Healthcare). Densitometry analysis was performed using ImageJ.

### In vitro activity/transcription assays with the purified IAV polymerase

To measure the ability of the IAV RNA polymerase to extend a capped primer, first a synthetic 11-nt-long RNA with 5′ diphosphate (ChemGenes; ppGAAUACUCAAG) was capped with a radiolabeled cap-1 structure in 20-μl reactions containing 1 μM RNA, 0.25 μM [α-^32^P]guanosine 5′-triphosphate (GTP) (3000 Ci/mmol, Revvity), 0.8 mM *S*-adenosylmethionine, *Vaccinia* virus (0.5 U/μl) capping enzyme (NEB), and 2′-*O*-methyltransferase (2.5 U/μl; NEB) at 37°C for 1 hour. The capped RNA was purified using an oligonucleotide cleanup kit (Zymo Research) and eluted in 30 μl of water. To test the transcriptional activity of the IAV RNA polymerase, we used 4-μl reactions containing 5 mM MgCl_2_, 1 mM DTT, RNase inhibitor (2 U/μl; APExBIO), 0.5 μM RNA template (table S3), 500 μM ATP, 500 μM cytidine 5′-triphosphate, 500 μM GTP, 0.2 μl capped RNA primer, 12.5% glycerol, 1% Tween 20, 100 mM NaCl, 25 mM Hepes (pH 8), and RNA polymerase (5 ng/μl). Reactions were incubated at 30°, 37°, and 39°C for 30 min and analyzed with 12% denaturing PAGE and autoradiography.

To measure the ability of the IAV RNA polymerase to cleave a capped primer, a synthetic 20-nt-long RNA with 5′ diphosphate (ChemGenes; ppAAUCUAUAAUAGCAUUAUCC) was first capped with a radiolabeled cap-1 structure as described above for the 11-nt-long primer. To test the cleavage activity, reactions (4 μl) were performed as described above, but NTPs were omitted and replaced with RNase-free water.

### RNA isolation

Total RNA was extracted from cells using TRIzol, chloroform method as previously described (*60*). RNA concentration was assessed using the Qubit 2 fluorometer (Thermo Fisher Scientific, USA) with Qubit RNA HS Assay Kit (Thermo Fisher Scientific, USA). The quality of total RNA expressed as RNA integrity number (RIN) was determined with Bioanalyzer 2100 instrument (Agilent, USA) using an Agilent RNA Pico 6000 Kit (Agilent, USA). The threshold RIN reading greater than 8.0 was taken as cutoff point for transition to the stage of library preparation.

### Library preparation and sequencing

A total of 18 cDNA libraries were prepared from three biological replicates of 12-hour mock and infected A549 and mock HEK293T cell line, respectively. Purified total RNA samples were first examined on Bioanalyzer 2100 using RNA 6000 Pico chip to evaluate the integrity and concentration (Agilent Technologies, CA) and then normalized onto 96-well plates. The poly(A) containing RNA transcripts in these samples were converted to cDNA using barcoded oligo-dT primers in the reverse transcription reaction to index each sample following the drop-seq method (*62*). The pooled barcoded cDNA samples were amplified by PCR and purified and then turned into sequencing libraries using the Illumina Tagment DNA Enzyme and Buffer kit (Illumina, CA) to include only the poly(A) tail adjacent 3′ ends of RNA transcripts. These libraries were examined on the Bioanalyzer (Agilent, CA) DNA HS chips for size distribution and quantified by Qubit fluorometer (Invitrogen, CA) and then sequenced on Illumina NovaSeq 6000 S Prime flowcell using the 100 cycle v1.5 kit. Raw sequencing reads were filtered by Illumina NovaSeq Control Software and only the Pass-Filter reads were used for further analysis.

### RNA-seq analysis

The raw data were saved as FASTQ format files. The quality control of the raw and trimmed reads was performed using fastp (*63*). The reads complementary to the genome of influenza A/WSN/1933 (H1N1) were filtered out from the trimmed reads, and the filtered reads were used for transcript quantification using RNA STAR tool (GRCh38_RefSeq_Transcripts) (*64*). edgeR was used to convert the transcript quantifications to gene quantifications (*65*).

### Differential gene expression analysis

To study genes involved in the cellular response to influenza A virus infection in A549 cells acclimated to 37° or 39°C and mock-infected HEK293T cells, DEGs between the temperatures were identified following a standard workflow using edgeR with an FDR-adjusted *P* value < 0.05 and the absolute value of a log_2_(fold change) > 1 (*65*). Reactome pathway enrichment analysis was used to categorize the top hits from the DEGs using R package ReactomePA (*66*).

### Flow cytometry

A549 cells were grown to near confluency in 12-well plates and subsequently infected with A/WSN/33 at an MOI of 3 or 1 in DMEM/0.5% fetal bovine serum (FBS) for 1 hour. The inoculum was then removed, and cells were incubated for 12 hours in DMEM/0.5% FBS at 37° or 39°C. For intracellular protein staining, cells were fixed and permeabilized using the eBioscience Foxp3/Transcription Factor Staining Buffer Set (Thermo Fisher Scientific) and next incubated with fluorescein isothiocyanate (FITC) Anti-Influenza A Virus Nucleoprotein antibody [D67J] (Abcam) for 1 hour at 4°C. The antibody was diluted 1:20 in permeabilization buffer before staining. Unstained and stained mock cells were used as a control. FITC fluorescence in infected and control cells was measured using a BD Biosciences FACSymphony A3 flow cytometer. The FITC was excited with a 488-nm laser, and fluorescence emission was collected via a 530/30 bandpass filter. Data were analyzed using FlowJo v9.4.11.

### TSO-based RT-PCR

For the template-switching oligo (TSO)-based RT-PCR, RNA was collected from A549 cells infected with an MOI of 1 of A/WSN/33 virus or mock infected. After extraction, 1 μg of RNA was either treated or mock-treated with oligo(dT)_25_ beads from the magnetic mRNA isolation kit (NEB) according to the manufacturer’s instructions and purified using the RNA Clean & Concentrator-5 kit (Zymo Research). Equal volumes (containing around 100 ng) of RNA were used for TSO-based RT. To this end, RNA was first denatured in the presence of dNTPs (1 mM final concentration) and either the Tuni-13 LNA3 (ACGCGTGATCAGTAGAAA+CA+AG+G) or the oligo(dT)_20_ primer (1 μM final concentration) in 3-μl volume at 70°C for 5 min. Denatured RNAs were immediately placed on ice for 5 min. Next, 2 μl of enzyme mix containing template switching RT buffer (NEB), template switching RT enzyme mix (NEB), and the TSO (GCTAATCATTGCAAGCAGTGGTATCAACGCAGAGTACATrGrGrG, 3.75 μM final concentration) were added to the RNA mix and incubated at 42°C for 90 min. Reactions were terminated by incubation at 85°C for 5 min and subsequently cooled to 4°C. The unused primers were digested with Thermolabile exonuclease I (NEB), and reactions were diluted 2× with water. One microliter of diluted RT reaction was used for PCR amplification with Q5 High-Fidelity DNA polymerase (NEB) using TSO-specific forward primer (CATTGCAAGCAGTGGTATCAAC) and either an NP-specific (GATTTCGATGTCACTCTGTGAG) or a PA-specific (GGATTGAAGCATTGTCGCAC) reverse primer. Thermocycling conditions included: (i) initial denaturation at 98°C for 30 s; (ii) 20 to 30 cycles of denaturation at 98°C for 10 s, annealing at 50°C for 15 s and extension at 72°C for 10 s; and (iii) final extension at 72°C for 2 min. Note that different numbers of cycles were used for 2- to 4-hour and 6- to 10-hour time points to avoid oversaturation. The PCR products were resolved on a 10% tris-borate EDTA acrylamide gel and visualized using SYBR Gold (Invitrogen).

### Statistical analysis

All data were represented as means ± SD. Data were analyzed in GraphPad Prism 10.0 software using Student’s *t* test. Statistical significance was set at a threshold of *P* < 0.05. Data are representative of *n* = 3 biological replicates, unless stated otherwise in the figure legends.
